# Poor neutralizing antibody responses against SARS‐CoV‐2 Omicron BQ.1.1 and XBB in Norway in October 2022

**DOI:** 10.1111/irv.13144

**Published:** 2023-06-02

**Authors:** Elisabeth Lea Vikse, Even Fossum, Magnhild Sekse Erdal, Olav Hungnes, Karoline Bragstad

**Affiliations:** ^1^ Department of Virology Norwegian Institute of Public Health Oslo Norway

**Keywords:** immune evasion, neutralizing antibodies, Omicron, SARS‐CoV‐2

## Abstract

New immune evasive variants of SARS‐CoV‐2 continue to emerge, potentially causing new waves of covid‐19 disease. Here, we evaluate levels of neutralizing antibodies against isolates of Omicron variants, including BQ.1.1 and XBB, in sera harvested 3–4 weeks after vaccination or breakthrough infections. In addition, we evaluate neutralizing antibodies in 32 sera from October 2022, to evaluate immunity in Norwegian donors prior to the winter season. Most serum samples harvested in October 2022 had low levels of neutralizing antibodies against BQ.1.1 and especially XBB, explaining why these variants and their descendants have dominated in Norway during the 2022 and 2023 winter season.

## 1 NEW OMICRON VARIANTS

New Omicron subvariants have demonstrated increased ability to evade immune responses induced by vaccination and/or infection,^1^, ^2^, ^3^ and resistance to existing monoclonal antibody treatments such as Evusheld (tixagevimab and cilgavimab) and bebtelovimab.^4^ Immune evasion has been linked to several mutations occurring in the receptor binding domain (RBD) of the surface glycoprotein (Spike), including amino acid (aa) positions R346, K444, L452, N460, and F486.^2^, ^5^, ^6^ Recently, sub‐lineages of both BA.2.75 and BA.5 have independently acquired substitutions in these aa positions, suggesting converging evolution and growth advantages relative to non‐mutated variants.^7^, ^8^ Whereas variants such as BA.2.75 and BA.5 contained one or two mutations in this group of aa positions, the newer BQ.1.1 strain (BA.5 derived) have acquired substitutions in all five aa positions. In addition, the recombination of two BA.2 derived lineages (BJ.1 and BM.1.1.1) has resulted in the formation of the XBB variant, which has proven highly immune evasive.^6^ The XBB variant contains substitutions in three of the RBD positions (R346, N460, and F486) in addition to a deletion in the N‐terminal domain of spike S1 (Y144).

Prior to the emergence of the Omicron variant in November 2021,^9^ infections with SARS‐CoV‐2 remained relatively low in Norway because of non‐pharmaceutical restrictions and a high vaccination coverage. In August 2021, a total of ~150.000 infections had been registered, corresponding to about 3% of the population. However, seroprevalence of IgG directed against nucleoprotein (N), an antigen not included in any of the vaccines used in Norway, was determined to be 11.7% in August 2021, suggesting that the number of infected was significantly higher.^10^ Nevertheless, most Norwegians remained uninfected prior to Omicron.

By the end of September 2022, >89% of Norwegians over 18 years had received at least two doses of covid‐19 vaccine, and >91% of those over 65 years had received at least three doses.^11^ Combined with the introduction of the Omicron variant in November 2021^12^ and the subsequent BA.1/BA.2 wave in January–March and BA.4/BA.5 wave in summer 2022, the vast majority of Norwegians has acquired immune responses against SARS‐CoV‐2. It is, however, uncertain how well these responses protect against novel Omicron variants. To remedy this situation, we evaluated neutralizing antibodies against isolates of Omicron variants in sera from recipients of three doses of monovalent mRNA vaccine and from individuals with BA.1, BA.2 or BA.5 breakthrough infections. In addition, we evaluate neutralizing antibodies against BA.5, BQ.1.1, and XBB in serum samples harvested from 32 individuals in October 2022.

## 2 IMMUNE EVASION OF NEW OMICRON VARIANTS

**FIGURE 1:**
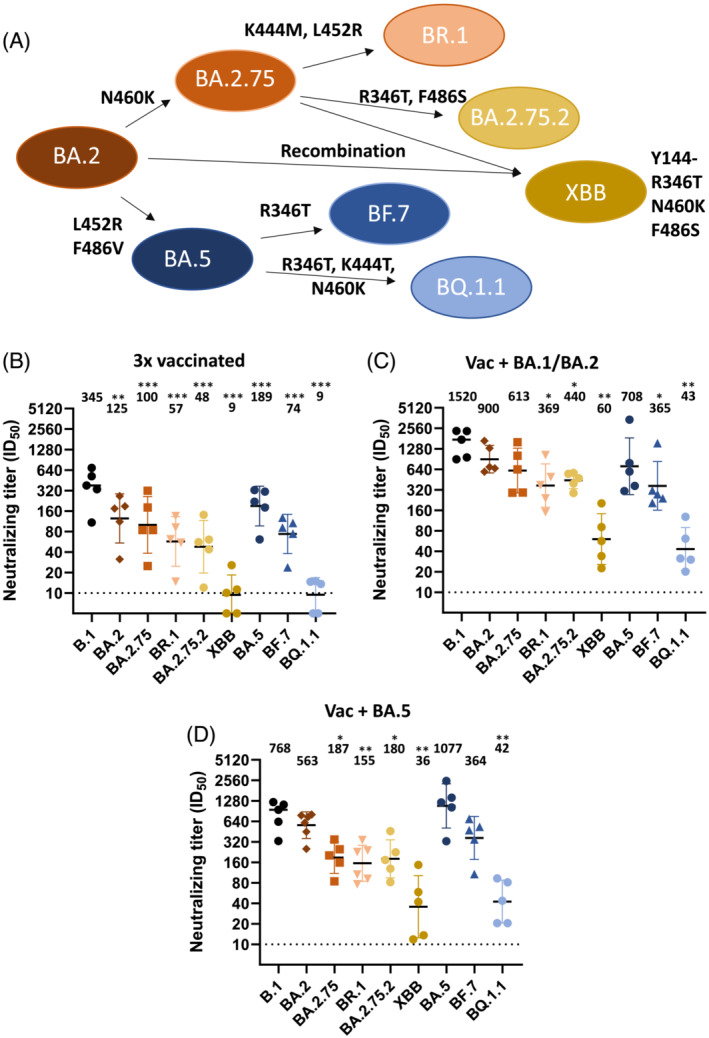
Omicron variants evade neutralizing antibodies induced by vaccination and breakthrough infection. (A) Overview of Omicron variants evaluated by neutralization assay, including the most relevant amino acid (aa) substitutions in the RBD of spike. (B–D) Neutralizations assays using sera from three times vaccinated individuals (B) without breakthrough infection, or from vaccinated individuals with breakthrough infection with (C) BA.1 or BA.2 or (D) BA.5. Geometric mean is indicated above each dataset. Data presented as geometric mean ± geometric standard deviation. Stippled line represents lowest dilution tested (10). N = 5 donors for each dataset. One‐way analysis of variance (ANOVA) compared with B.1 with Dunnett's multiple comparison test, *p < 0.05; **p < 0.01; ***p < 0.001.

Throughout the pandemic, SARS‐CoV‐2 specimens have been genetically characterized by whole genome sequencing as part of the Norwegian covid‐19 surveillance effort. To evaluate neutralizing antibodies against variants of interest, isolates of Omicron variants BA.2, BA.5, BA.2.75, BA.2.75.2, BF.7, BR.1, BQ.1.1, and XBB were grown in VeroE6/TMPRSS2 or Vero/hSLAM cells (Figure 1A, Supplementary Table S1, and Supplementary Data S1). For comparison we also included the B.1 strain that was isolated in April 2020 that only contains the spike mutation D614G. Virus isolates were passaged twice, and the second passage was used for the subsequent neutralization assays. The passaged virus was sequenced to ensure the genotype of the variant. Although we observed a limited number of nucleotide substitutions in the passaged viruses, none led to aa changes in the Spike protein.

Sera from three times mRNA vaccinated individuals and from two‐ or three‐times vaccinated individuals with subsequent breakthrough infections with BA1, BA.2, or BA.5 were analyzed for the ability to neutralize the viral isolates (Supplementary Table S2). Serum was harvested 3–4 weeks after vaccination or breakthrough infection. Whereas sera from three times vaccinated individuals efficiently neutralized B.1, there was a significant reduction in neutralizing titers against the other strains. We still observed moderate neutralization of BA.2, BA.2.75, and BA.5; whereas, there was hardly any neutralization of BQ.1.1 and XBB (Figure 1B).

Sera from people with breakthrough infections with BA.1, BA.2, or BA.5 generally had higher neutralizing titers against all tested variants, compared with vaccinated‐only individuals (Figure 1C,D). As expected, people with BA.5 breakthrough infection had the highest neutralizing titers against BA.5 virus, although not significantly higher than against B.1 (Figure 1D). In contrast, people with BA.1 or BA.2 breakthrough infections had the highest neutralizing titers against the B.1 strain, although the titers were not significantly different from BA.2, BA.2.75, and BA.5. People with BA.1 or BA.2 infection had strong neutralizing titers against more recent variants such as BA.2.75, BR.1, BA.2.75.2, and BF.7, although significantly lower than against B.1 (Figure 1C). There was, however, a marked and significant reduction in neutralizing antibodies against the BQ.1.1 and XBB also in people with breakthrough infection (Figure 1C,D).

## 3 NEUTRALIZING ANTIBODIES AGAINST BQ.1.1 AND XBB IN OCTOBER 2022

**FIGURE 2:**
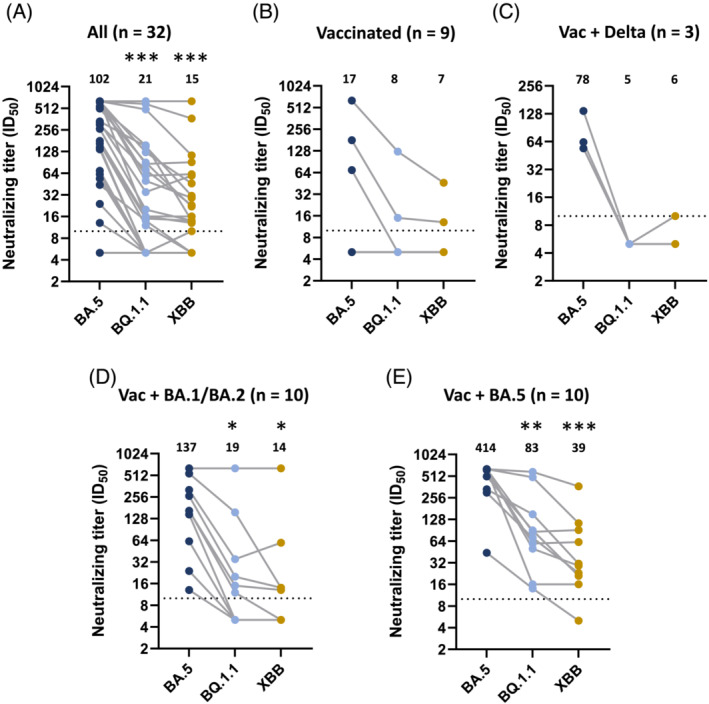
Neutralizing antibodies towards BQ.1.1 and XBB in sera collected October 2022. (A) Sera from 32 donors were evaluated in neutralization assays against Omicron variants BA.5, BQ.1.1 and XBB. (B–D) Sera from (A) were divided into groups of (B) vaccinated without infection (n = 9), (C) vaccinated with Delta breakthrough infection (Vac + Delta, n = 3), (D) vaccinated with BA.1 or BA.2 breakthrough infection (Vac + BA.1/BA.2, n = 10), or (E) vaccinated with BA.5 breakthrough infection (Vac + BA.5 n = 10) and evaluated for neutralizing antibodies against BA.5, BQ.1.1, or XBB. Geometric mean is indicated above each dataset. Data presented as geometric mean ± geometric standard deviation. Stippled line represents lowest dilution tested (10). (A) n = 32, and (B–E) n = 3–10 donors for each dataset. Matched one‐way analysis of variance (ANOVA) with Tukey's multiple comparison test. *p < 0.05; **p < 0.01; ***p < 0.001.

Serum samples taken shortly after vaccination or breakthrough infection may not necessarily provide a good picture of the current levels of neutralizing antibodies. To evaluate immunity towards the more immune evasive Omicron variants prior to the winter season, 32 serum samples were harvested from healthy individuals on October 13–14, 2022 and evaluated for neutralizing titers against BA.5, BQ.1.1, and XBB. The average age of the donors were 42.6 years, and the donors consisted of 78% females. Whereas neutralizing titers against BA.5 remained elevated (≥64) in 20 of 32 tested individuals, there was a significant reduction in neutralizing antibodies against BQ.1.1 and especially XBB (Figure 2A). For BQ.1.1, 9 of 32 had neutralizing titers >64, and only 4 of 32 had titers >64 against XBB.

Next, we divided the donors into groups based on vaccination and infection history (Supplementary Table S3). It should be noted that donors may have undergone mild or subclinical infections that have not been accounted for. In general, presumed uninfected donors had lower titers of neutralizing antibodies against all tested variants (Figure 2B). One uninfected donor (#32) had recently received a 4th vaccine dose and had the highest titers against all variants in this group. Donors with previous breakthrough infections with BA.1 or BA.2 and especially BA.5, had high neutralizing titers towards BA.5. Interestingly, the three donors with only Delta breakthrough also had reasonably high titers against BA.5 (Figure 2C). There were no non‐vaccinated individuals among the donors.

There was a significant reduction in neutralizing antibodies against BQ.1.1 and XBB in donors with BA.1, BA.2, or BA.5 breakthrough infections (Figure 2D,E). However, 6/10 donor with BA.5 breakthrough infection maintained neutralizing titers >64 (Figure 2E), which may provide some protection from infection.^13^ People with BA.5 breakthrough had higher neutralizing titers against BQ.1.1 and XBB, than people with BA.1 or BA.2 breakthrough, but this is likely due to the shorter time since infection.

## 4 DISCUSSION

Our observations corroborate the immune evasive nature of the BQ.1.1 and XBB variant as observed by others.^6^, ^7^ When evaluating sera harvested 3–4 weeks after the third vaccination or after breakthrough infection, neutralizing antibody levels were reduced for most strains compared with B.1, although the reduction was most prominent against BQ.1.1 and XBB. Donors with breakthrough infection of BA.1 or BA.2 had slightly higher neutralizing titers against XBB than BQ.1.1, which is in contrast to what others have observed.^6^ When evaluating neutralizing titers in sera harvested in October 2022, donors with breakthrough infections with BA.5 had higher neutralizing titers against both BQ.1.1 and XBB compared with donors with BA.1 or BA.2 breakthrough. However, this likely reflects the more recent BA.5 wave of infections and not that BA.5 infection induces neutralizing antibodies with broader specificity. Indeed, sera harvested 3–4 weeks after BA.1 or BA.2 infection neutralized BA.2.75 derived variants (BR.1 and BA.2.75.2) and BA.5 derived variant (BF.7) equally well; whereas, sera from BA.5 breakthrough neutralized BF.7 better than BR.1 and BA.2.75.2 (Figure 1).

Although neutralizing antibodies have been established as a correlate of protection against SARS‐CoV‐2,^13^, ^14^, ^15^ it is less clear what constitutes protective levels of neutralizing antibodies. Gilbert and colleagues observed a 91% risk‐reduction of covid‐19 (symptomatic infection) with neutralizing titer of 100 in a pseudotype assay during a 100‐days follow‐up period.^14^ Similarly, Dimeglio and colleagues observed 94% reduced risk of infection (symptomatic and asymptomatic) with a neutralizing titer of 64–128 using live virus, over a follow‐up period of 275 days.^13^ Whereas protective efficacy cannot be extrapolated from these studies onto our data, 9/32 donors had neutralizing antibody titers over 64 against BQ.1.1, including 6/10 donors with BA.5 breakthrough infections. It therefore seems plausible that BA.5 breakthrough infections have conferred some degree of protection against BQ.1.1. In contrast, only 4/32 donors had neutralizing titers above 64 against the XBB variant.

## 5 CONCLUSION

We observed a marked reduction in neutralizing antibodies against the immune evasive Omicron variants BQ.1.1 and XBB in serum samples from October 2022. Nevertheless, people with BA.5 breakthrough infections likely retained some protection against the variant, especially BQ.1.1. The fact that immunity against BA.1 and BA.2 has waned over several months, combined with lower neutralizing titers against XBB (a BA.2 recombinant), may explain why XBB and subvariants have become dominant during the first months of 2023.^11^

## AUTHOR CONTRIBUTIONS

Elisabeth Lea Vikse developed the methodology, performed the experiments, analyzed the results, conceptualized the study, and reviewed and edited the final manuscript. Magnhild Sekse Erdal performed the experiments, analyzed the results, and reviewed and edited the final manuscript. Even Fossum performed the experiments, analyzed the results, administered and supervised the project, wrote the first draft, and reviewed and edited the final manuscript. Olav Hungnes conceptualized the study, administered and supervised the project, and reviewed and edited the final manuscript. Karoline Bragstad conceptualized the study, administered and supervised the project, and reviewed and edited the final manuscript.

## CONFLICT OF INTEREST STATEMENT

The authors report no financial or other conflict of interest.

### ETHICAL STATEMENT

A separate ethical approval was not obtained, as this study was a part of the routine surveillance and investigation of virus properties performed at NIPH as the national reference laboratory for coronaviruses with outbreak potential. NIPH is authorized to conduct these studies under the Norwegian Infection control act and the regulations relating to notifiable diseases. All human sera were obtained with written consent.

### PEER REVIEW

The peer review history for this article is available at https://www.webofscience.com/api/gateway/wos/peer-review/10.1111/irv.13144.

## Supporting information


**Table S1.** Viral isolates used for neutralization assay. Virus genome sequences (original specimen) are available in GISAID EpiCoV with accession numbers EPI_ISL_449791 (B.1), EPI_ISL_12981999 (BA.5), EPI_ISL_16100571 (BA.2), EPI_ISL_14773262 (BF.7), EPI_ISL_14892153 (BA.2.75), EPI_ISL_15191765 (BR.1), EPI_ISL_15349765 (BQ.1.1), and EPI_ISL_15538637 (XBB).

**Table S2.** Characterization of sera used in neutralization assays against different Omicron strains in Figure 1.

**Table S3.** Neutralizing titers against BA.5, BQ.1.1 and XBB in sera collected from healthy donors in October 2022. For calculations, titers below the minimal dilution of 10 were plotted as 5, and titers at or above the maximal dilution of 640 were plotted as 640.

**Data S1.** Supporting Information

## Data Availability

Anonymized data can be shared in accordance with the data sharing policy of NIPH. Virus genome sequences (original specimen) are available in GISAID EpiCoV with accession numbers EPI_ISL_449791 (B.1), EPI_ISL_12981999 (BA.5), EPI_ISL_16100571 (BA.2), EPI_ISL_14773262 (BF.7), EPI_ISL_14892153 (BA.2.75), EPI_ISL_15191765 (BR.1), EPI_ISL_15349765 (BQ.1.1), and EPI_ISL_15538637 (XBB).

## References

[1] Kurhade C , Zou J , Xia H , et al. Low neutralization of SARS‐CoV‐2 Omicron BA.2.75.2, BQ.1.1, and XBB.1 by parental mRNA vaccine or a BA.5‐bivalent booster. Nat Med. 2022;29:344‐347. 36473500 10.1038/s41591-022-02162-x

[2] Cao Y , Yisimayi A , Jian F , et al. BA.2.12.1, BA.4 and BA.5 escape antibodies elicited by omicron infection. Nature. 2022;608(7923):593‐602. doi:10.1038/s41586-022-04980-y 35714668 PMC9385493

[3] Davis‐Gardner ME , Lai L , Wali B , et al. Neutralization against BA.2.75.2, BQ.1.1, and XBB from mRNA bivalent booster. N Engl J Med. 2022;388(2):183‐185. doi:10.1056/NEJMc2214293 36546661 PMC9812288

[4] Arora P , Kempf A , Nehlmeier I , et al. Omicron sublineage BQ.1.1 resistance to monoclonal antibodies. Lancet Infect Dis. 2022;23(1):22‐23. doi:10.1016/S1473-3099(22)00733-2 36410372 PMC9707647

[5] Planas D , Bruel T , Staropoli I , et al., Resistance of Omicron subvariants BA.2.75.2, BA.4.6 and BQ.1.1 to neutralizing antibodies. bioRxiv, 2022. 10.1038/s41467-023-36561-6 PMC9926440 36788246

[6] Uraki R , Ito M , Furusawa Y , et al. Humoral immune evasion of the omicron subvariants BQ.1.1 and XBB. Lancet Infect Dis. 2022;23(1):30‐32. doi:10.1016/S1473-3099(22)00816-7 36495917 PMC9729000

[7] Cao Y , Jian F , Wang J , et al. Imprinted SARS‐CoV‐2 humoral immunity induces convergent Omicron RBD evolution. Nature. 2022;614:521‐529. doi:10.1038/s41586-022-05644-7 36535326 PMC9931576

[8] Ito J , Suzuki R , Uriu K , et al., Convergent evolution of the SARS‐CoV‐2 Omicron subvariants leading to the emergence of BQ.1.1 variant. bioRxiv, 2022. doi:10.1101/2022.12.05.519085 PMC10175283 37169744

[9] Karim SSA , Karim QA . Omicron SARS‐CoV‐2 variant: a new chapter in the COVID‐19 pandemic. Lancet. 2021;398(10317):2126‐2128. doi:10.1016/S0140-6736(21)02758-6 34871545 PMC8640673

[10] Tunheim G , Ro GOI , Chopra A , et al. Prevalence of antibodies against SARS‐CoV‐2 in the Norwegian population, august 2021. Influenza Other Respi Viruses. 2022;16(6):1004‐1013. doi:10.1111/irv.13024 PMC9349429 35770841

[11] The Norwegian Institute of Public Health . Health NIoP, Weekly infection report, in Weekly infection report. 2022. https://www.fhi.no/publ/2020/koronavirus-ukerapporter/

[12] Brandal LT , MacDonald E , Veneti L , et al. Outbreak caused by the SARS‐CoV‐2 Omicron variant in Norway, November to December 2021. Euro Surveill. 2021;26(50):2101147. doi:10.2807/1560-7917.ES.2021.26.50.2101147 34915975 PMC8728491

[13] Dimeglio C , Herin F , Martin‐Blondel G , Miedouge M , Izopet J . Antibody titers and protection against a SARS‐CoV‐2 infection. J Infect. 2022;84(2):248‐288. doi:10.1016/j.jinf.2021.09.013 PMC8452591 34560135

[14] Gilbert PB , Montefiori DC , McDermott AB , et al. Immune correlates analysis of the mRNA‐1273 COVID‐19 vaccine efficacy clinical trial. Science. 2022;375(6576):43‐50. doi:10.1126/science.abm3425 34812653 PMC9017870

[15] Khoury DS , Cromer D , Reynaldi A , et al. Neutralizing antibody levels are highly predictive of immune protection from symptomatic SARS‐CoV‐2 infection. Nat Med. 2021;27(7):1205‐1211. doi:10.1038/s41591-021-01377-8 34002089

