# Supplementary material for: Poor neutralizing antibody responses against SARS‐CoV‐2 Omicron BQ.1.1 and XBB in Norway in October 2022
Source: Influenza Other Respir Viruses. 2023 Jun 2;17(6):e13144. doi: 10.1111/irv.13144 (PMC10236499; doi:10.1111/irv.13144)
Supplement: Supplementary file 1 — Table S1. Viral isolates used for neutralization assay. Virus genome sequences (original specimen) are available in GISAID EpiCoV with accession numbers EPI_ISL_449791 (B.1), EPI_ISL_12981999 (BA.5), EPI_ISL_16100571 (BA.2), EPI_ISL_14773262 (BF.7), EPI_ISL_14892153 (BA.2.75), EPI_ISL_15191765 (BR.1), EPI_ISL_15349765 (BQ.1.1), and EPI_ISL_15538637 (XBB). [file IRV-17-e13144-s004.docx]

**Supplementary table I:** Viral isolates used for neutralization assay. Virus genome sequences (original specimen) are available in GISAID EpiCoV with accession numbers EPI_ISL_449791 (B.1), EPI_ISL_12981999 (BA.5), EPI_ISL_16100571 (BA.2), EPI_ISL_14773262 (BF.7), EPI_ISL_14892153 (BA.2.75), EPI_ISL_15191765 (BR.1), EPI_ISL_15349765 (BQ.1.1), and EPI_ISL_15538637 (XBB).

| **Isolate** | **Sample date** | **Pangolin variant** | **Defining mutations in RBD** | **Full list of mutations in the Spike protein, relative to nCoV2019/Wuhan-Hu-1/MN908947** |
| --- | --- | --- | --- | --- |
| hCoV-19/Norway/2386/2020 | 04.04.20 | B.1 | D614G | D614G |
| SC2/Norway/00126/2022 | 02.01.2022 | BA.2 | Reference | T19I;L24S;G142D;V213G;G339D;S371F;S373P;S375F;T376A;D405N;R408S;K417N;N440K;S477N;T478K;E484A;Q493R;Q498R;N501Y;Y505H;D614G;H655Y;N679K;P681H;N764K;D796Y;Q954H;N969K;P25-;P26-;A27- |
| SC2/Norway/21334/2022 | 02.05.2022 | BA.5 | L452R, F486V | T19I;L24S;G142D;V213G;G339D;S371F;S373P;S375F;T376A;D405N;R408S;K417N;N440K;L452R;S477N;T478K;E484A;F486V;Q498R;N501Y;Y505H;D614G;H655Y;N679K;P681H;N764K;D796Y;Q954H;N969K;D1139Y;P25-;P26-;A27-;H69-;V70- |
| SC2/Norway/29067/2022 | 31.07.2022 | BF.7 | R346T, L452R, F486V | T19I;L24S;G142D;V213G;G339D;R346T;S371F;S373P;S375F;T376A;D405N;R408S;K417N;N440K;L452R;S477N;T478K;E484A;F486V;Q498R;N501Y;Y505H;D614G;H655Y;N679K;P681H;N764K;D796Y;Q954H;N969K;P25-;P26-;A27-;H69-;V70- |
| SC2/Norway/30134/2022 | 16.08.2022 | BA.2.75 | N460K | T19I;L24S;G142D;K147E;W152R;F157L;I210V;V213G;G257S;G339H;K356T;S371F;S373P;S375F;T376A;D405N;R408S;K417N;N440K;G446S;N460K;S477N;T478K;E484A;Q498R;N501Y;Y505H;D614G;H655Y;N679K;P681H;N764K;D796Y;Q954H;N969K;P25-;P26-;A27- |
| SC2/Norway/32397/2022 | 14.09.2022 | BA.2.75.2 | R346T, N460K, F486S | T19I;L24S;G142D;K147E;W152R;F157L;I210V;V213G;G257S;G339H;R346T;S371F;S373P;S375F;T376A;D405N;R408S;K417N;N440K;G446S;N460K;S477N;T478K;E484A;F486S;Q498R;N501Y;Y505H;D614G;H655Y;N679K;P681H;N764K;D796Y;Q954H;N969K;D1199N;P25-;P26-;A27- |
| SC2/Norway/31280/2022 | 09.09.2022 | BR.1 | K444M, L452R, N460K | T19I;L24S;G142D;K147E;W152R;F157L;I210V;V213G;G257S;G339H;S371F;S373P;S375F;T376A;D405N;R408S;K417N;N440K;K444M;G446S;L452R;N460K;S477N;T478K;E484A;Q498R;N501Y;Y505H;D614G;H655Y;N679K;P681H;N764K;D796Y;Q954H;N969K;P25-;P26-;A27- |
| SC2/Norway/31371/2022 | 15.09.2022 | BQ.1.1 | R346T, K444T, L452R, N460K, F486V | T19I;L24S;G142D;V213G;G339D;R346T;S371F;S373P;S375F;T376A;D405N;R408S;N440K;K444T;L452R;N460K;S477N;T478K;E484A;F486V;Q498R;N501Y;Y505H;D614G;H655Y;N679K;P681H;N764K;D796Y;Q954H;N969K;P25-;P26-;A27-;H69-;V70- |
| SC2/Norway/32121/2022 | 05.10.2022 | XBB | Y144-, R346T, N460K, F486S | T19I;L24S;V83A;G142D;H146Q;Q183E;V213E;G339H;R346T;L368I;S371F;S373P;S375F;T376A;D405N;R408S;N440K;V445P;G446S;N460K;S477N;T478K;E484A;F486S;F490S;Q498R;N501Y;Y505H;D614G;H655Y;N679K;P681H;N764K;D796Y;Q954H;N969K;P25-;P26-;A27-;Y144- |
