# Supplementary material for: Poor neutralizing antibody responses against SARS‐CoV‐2 Omicron BQ.1.1 and XBB in Norway in October 2022
Source: Influenza Other Respir Viruses. 2023 Jun 2;17(6):e13144. doi: 10.1111/irv.13144 (PMC10236499; doi:10.1111/irv.13144)
Supplement: Supplementary file 2 — Table S2. Characterization of sera used in neutralization assays against different Omicron strains in Figure 1. [file IRV-17-e13144-s002.docx]

| **Donors** | **Sex (M/F)** | **Age** | **SARS-CoV-2 infection** |
| --- | --- | --- | --- |
|  | F | 62 | None |
|  | F | 26 | None |
|  | F | 28 | None |
|  | F | 42 | None |
|  | F | 27 | None |
|  | F | 38 | BA.2 |
|  | F | 42 | BA.1 |
|  | F | 62 | BA.2 |
|  | F | 26 | BA.2 |
|  | F | 34 | BA.1 |
|  | F | 29 | BA.5 |
|  | F | 28 | BA.5 |
|  | F | 28 | BA.5 |
|  | F | 52 | BA.5 |
|  | F | 30 | BA.5 |

Supplementary table II: Characterization of sera used in neutralization assays against different Omicron strains in Figure 1.
