# Supplementary material for: Poor neutralizing antibody responses against SARS‐CoV‐2 Omicron BQ.1.1 and XBB in Norway in October 2022
Source: Influenza Other Respir Viruses. 2023 Jun 2;17(6):e13144. doi: 10.1111/irv.13144 (PMC10236499; doi:10.1111/irv.13144)
Supplement: Supplementary file 3 — Table S3. Neutralizing titers against BA.5, BQ.1.1 and XBB in sera collected from healthy donors in October 2022. For calculations, titers below the minimal dilution of 10 were plotted as 5, and titers at or above the maximal dilution of 640 were plotted as 640. [file IRV-17-e13144-s001.docx]

**Supplementary table III:** Neutralizing titers against BA.5, BQ.1.1 and XBB in sera collected from healthy donors in October 2022. For calculations, titers below the minimal dilution of 10 were plotted as 5, and titers at or above the maximal dilution of 640 were plotted as 640.

| **Donors** | **Sex (M/F)** | **Age** | **SARS-CoV-2 infection** | **# of vaccine doses** | **BA.5**  **ID**_50_ | **BQ.1.1 ID**_50_ | **XBB**  **ID_50_** |
| --- | --- | --- | --- | --- | --- | --- | --- |
| 1 | F | 32 | None | 3 | <10 | <10 | <10 |
| 2 | F | 46 | Delta | 2 | 137 | <10 | <10 |
| 3 | F | 44 | None | 3 | <10 | <10 | <10 |
| 4 | F | 30 | BA.5 | 3 | ≥640 | 16 | 16 |
| 5 | M | 38 | None | 3 | <10 | <10 | <10 |
| 6 | F | 56 | BA.5 | 3 | 44 | 14 | <10 |
| 7 | F | 36 | Delta | 2 | 54 | <10 | 10 |
| 8 | F | 26 | None | 3 | 69 | <10 | 10 |
| 9 | F | 29 | BA.5 | 3 | ≥640 | 50 | 29 |
| 10 | F | 54 | BA.5 | 3 | ≥640 | 494 | 113 |
| 11 | M | 45 | BA.1/2 | 3 | 264 | 20 | 14 |
| 12 | F | 28 | BA.5 | 3 | 336 | 151 | 31 |
| 13 | F | 63 | BA.2 | 3 | 165 | 15 | 14 |
| 14 | F | 40 | None | 3 | 180 | 16 | 13 |
| 15 | M | 51 | BA.5 | 3 | 301 | 71 | 23 |
| 16 | F | 58 | None | 3 | <10 | <10 | <10 |
| 17 | F | 36 | BA.1 | 2 | 146 | <10 | <10 |
| 18 | M | 38 | BA.1/2 | 3 | 62 | <10 | <10 |
| 19 | F | 28 | BA.5 | 3 | ≥640 | 86 | 91 |
| 20 | F | 32 | BA.1/2 | 3 | 166 | 12 | <10 |
| 21 | F | 33 | BA.1/2 | 2 | 13 | <10 | <10 |
| 22 | F | 52 | BA.5 | 3 | 621 | 59 | 62 |
| 23 | M | 62 | BA.5 | 3 | 505 | 90 | 22 |
| 24 | F | 54 | None | 3 | <10 | <10 | <10 |
| 25 | F | 35 | BA.1 | 2 | ≥640 | ≥640 | ≥640 |
| 26 | F | 47 | Delta | 3 | 63 | <10 | <10 |
| 27 | F | 50 | None | 3 | <10 | <10 | <10 |
| 28 | F | 51 | BA.5 | 3 | ≥640 | 587 | 369 |
| 29 | M | 56 | BA.1/2 | 3 | 542 | 156 | 14 |
| 30 | F | 28 | BA.1/2 | 3 | 24 | <10 | <10 |
| 31 | F | 46 | BA.1/2 | 3 | 320 | 35 | 59 |
| 32 | M | 39 | None | 4 | ≥640 | 125 | 46 |
