# Supplementary material for: Poor neutralizing antibody responses against SARS‐CoV‐2 Omicron BQ.1.1 and XBB in Norway in October 2022
Source: Influenza Other Respir Viruses. 2023 Jun 2;17(6):e13144. doi: 10.1111/irv.13144 (PMC10236499; doi:10.1111/irv.13144)
Supplement: Supplementary file 4 — Data S1. Supporting Information [file IRV-17-e13144-s003.docx]

**Supplementary Materials**

**Material and Methods**

*Sera from donors*

Sera was collected from employees at the Norwegian Institute of Public health. All samples were collected with written consent. For Figure 1, sera were harvested 3-4 weeks after vaccination or breakthrough infection with a confirmed BA.1, BA.2 or BA.5 infection (Supplementary Table II). For sera harvested in October 2022, donors were defined as BA.1 or BA.2 breakthrough if they had been infected in January-March, and BA.5 infected if they had been infected during May-August (Supplemetary Table III). All previously uninfected individuals were fully vaccinated (3 doses of mRNA vaccines), and one donor had received a 4^th^ dose with a bivalent mRNA vaccine. It should be noted that donors may have undergone mild or subclinical infections which have not been accounted for in the groups.

*Virus isolation and titration*

Vero/hSLAM (ECACC #04091501) and Vero E6/TMPRSS2 (NIBSC #100978) were cultivated in complete Dulbecco’s Modified Eagle Medium (cDMEM) supplemented with 10% fetal bovine serum (FBS) and 0,4mg/ml (Vero/hSLAM) or 1mg/ml (VeroE6/TMPRSS2) G418.

In a biosafety level 3 (BSL3) facility, viral specimens collected from SARS-CoV-2 infected patients in Norway and selected based on the viral genome sequence were incubated on Vero/hSLAM or Vero E6/TMPRSS2 cells at approx. 60% confluency for 1 hour at 37°C. After incubation the inoculate was removed and replaced with fresh viral culture medium (DMEM supplemented with 2% FBS, 100 units/ml penicillin, 100 ug/ml streptomycin and 25 mM HEPES). The cells were incubated for 3-4 days at 37 °C and the supernatant was then diluted 1:1000 and passaged onto fresh cells. After 3-4 more days the second passage of virus was harvested and frozen in aliquots. The viral stocks were analyzed by qRT-PCR and sequenced by Illumina NGS to confirm the variant sequence and to ensure that no critical mutations had been selected for during the virus cultivation in cells. The harvested virus was titrated on Vero E6 cells to determine the tissue culture infectious dose (TCID_50_) before the virus neutralization assay was performed.

*Neutralization assay*

The neutralization assay and ELISA protocol was adapted and modified from Amanat et al. [1]. Vero E6 cells were cultivated in Minimum essential medium (MEM) supplemented with 5% FBS. Post-exposure sera were collected three to four weeks post vaccination or infection. In addition, we collected sera from 32 individuals in October 2022 from both fully vaccinated non-infected individuals and individuals with previous breakthrough infections of various SARS-CoV-2 variants. The negative serum control used was a pool of sera collected in early 2019. The sera were heat inactivated for 30 minutes at 56 °C and serially diluted twofold in a 96 well deep well plate in virus diluent (MEM supplemented with 2% FBS, 100 units/ml penicillin, 100 ug/ml streptomycin and 25 mM HEPES). In a BSL-3 facility, a viral dose of 100xTCID_50_ was added to each well of diluted sera and to a virus control not containing serum. Cell controls only containing virus diluent were also included. The virus-serum mix was incubated at 37°C for 1h and then added to a 96 well plate of Vero E6 cells with 12000 cells per well seeded one day prior. After 96 hours of incubation at 37°C the plates were checked for cytopathic effect using a light microscope. The cells were fixed with 80% acetone, and the plates transported out of the BSL3 facility.

An ELISA detecting the nucleocapsid of SARS-CoV-2 was performed on the fixed cell layer in a BSL2 facility. The ELISA consists of a blocking step using PBS with 1% BSA, primary incubation with SARS-CoV-2 nucleocapsid antibody (Sino biological, 40143-R019) and secondary incubation with Goat anti-rabbit IgG Alkaline Phosphatase Antibody (Sigma, A3687). Between each incubation step the plates were washed with wash buffer (PBS with 2% Tween 20). Finally, 1 mg/mL of phosphatase substrate dissolved in diethanolamine buffer was added to the plates, and the OD_405_ was measured for each well after 40 minutes. The 50% virus neutralization titer was calculated from the measured OD values using Graph-Pad PRISM version 9.

To quality control our neutralization assay, the B.1 strains has been evaluated against a reagent from the WHO International Reference Panel (20/130) which yielded a 50% neutralizing titer of 347 [2].

*Statistics*

Neutralization data is presented as geometric mean with geometric standard deviation. One-way ANOVA Tukey`s multiple comparison test was used to compare data sets. All statistics were calculated using Graph-Pad PRISM version 9.

**References**

1. Amanat F, Stadlbauer D, Strohmeier S, Nguyen THO, Chromikova V, McMahon M, et al., *A serological assay to detect SARS-CoV-2 seroconversion in humans.* Nat Med, 2020. **26**(7): p. 1033-1036.

2. Knezevic I, Mattiuzzo G, Page M, Minor P, Griffiths E, Nuebling M, et al., *WHO International Standard for evaluation of the antibody response to COVID-19 vaccines: call for urgent action by the scientific community.* Lancet Microbe, 2022. **3**(3): p. e235-e240.
